# Immune Infiltrates of m5C RNA Methylation-Related LncRNAs in Uterine Corpus Endometrial Carcinoma

**DOI:** 10.1155/2022/1531474

**Published:** 2022-03-29

**Authors:** Wen-Xiu Gu, Yan Chen, Wei Wang

**Affiliations:** ^1^Liyang People's Hospital, Liyang, Changzhou 213399, China; ^2^Jiangsu Province Geriatric Hospital, Nanjing 210029, China

## Abstract

Aberrant 5-methylcytidine (m5C) modification plays an essential role in the progression of different cancers. More and more researchers are focusing on developing a lncRNA-based risk model to assess the clinical prognosis of cancer patients. However, the impact of m5C-related lncRNAs on the prognosis of patients with uterine corpus endometrial carcinoma (UCEC), as well as the immune microenvironment of UCEC, remains unclear. Here, we comprehensively analyzed the predictive value of m5C-associated lncRNAs in UCEC and their association with the tumor immune microenvironment, according to the information extracted from the TCGA-UCEC dataset. We identified a total of 32 m5C-associated lncRNAs that were significantly correlated with the prognosis of UCEC patients. Two molecular subtypes were determined by consensus clustering analysis of these 32 m5C-associated prognostic lncRNAs. Further data showed that cluster 1 was associated with poor clinical prognosis, advanced tumor grade, higher PD-L1 expression levels, higher ESTIMATEScore, and higher immuneScore, as well as the immune cell infiltration. Then, 17 m5C-associated lncRNAs with prognostic values were obtained using LASSO regression analysis. And a risk model was constructed based on these 17 lncRNAs. It was revealed that the risk model could be used as an independent factor for UCEC prognosis. In addition, patients with UCEC in the high-risk group had higher tumor grades and immune scores. The risk model based on m5C-related lncRNAs was also closely associated with infiltrating immune cells. In conclusion, our study elucidated the crucial roles of the identified m5C-related lncRNAs in the UCEC patients' prognoses, as well as in the immune microenvironment in UCEC. The results suggest that the components of risk models based on the m5C-related lncRNAs may serve as important mediators of the immune microenvironment in UCEC.

## 1. Introduction

Uterine corpus endometrial carcinoma (UCEC) is one of the most common gynecologic tumors [1]. More than 50,000 women worldwide die from UCEC each year [2]. Currently, surgery combined with radiotherapy and/or chemotherapy is the standard clinical treatment option for patients with UCEC [3]. Despite the rapid development of modern medicine, the mortality rate of UCEC has continued to increase during recent years, and the prognosis of UCEC patients varies [4]. Several clinical features of UCEC patients and some molecular biomarkers have been used to predict the clinical prognosis, but these methods have limitations. Therefore, there is a need to construct a new predictive risk model to predict the prognosis of patients with UCEC and to identify new prognostic markers for UCEC.

5-Methylcytidine (m5C) is a modification that occurs on DNA and RNA [5], and it plays a nonnegligible role in various biological processes [6]. With the development of technologies such as high-throughput sequencing, it has become less challenging to identify and quantify m5C modifications in low-abundance RNA species, such as those on mRNAs and long noncoding RNAs (lncRNAs) [7]. A recent study specifies that m5C sites are predominantly enriched in the CG of mRNAs, reflecting the tissue-specific and dynamic nature of m5C in the mammalian transcriptome [8]. So far, researchers have explored the distribution and function of m5C modifications in a number of different types of RNAs [9, 10]. However, studies on the prevalence and distribution of m5C in lncRNAs are still very few.

LncRNAs are a class of RNA molecules that contain over 200 nucleotides in length, and they are mainly derived from the noncoding regions of the genome [11, 12]. In recent years, lncRNAs have gained widespread attention as key regulators in various physiological and pathological processes [13]. And epigenetic regulation of lncRNAs is one of the main mechanisms controlling their expression and tissue specificity. RNA methylation, as one of the important epigenetic modifications, has been identified as an important marker of tumorigenesis [14, 15]. In addition, studies have shown that m5C modifications are also closely associated with immune cell infiltration [6]. However, the functions and mechanisms of m5C-associated lncRNAs in cancer remain enigmatic. Therefore, it is important to explore m5C-associated lncRNAs and clarify biomarkers of prognostic value in these lncRNAs.

The programmed death-ligand 1 (PD-L1) has been reported upregulated in various tumors [16]. Programmed cell death protein 1 (PD-1), the receptor for PD-L1, regulates the effector T cell responses in vivo, which is closely related to the immune suppression in tumors [17]. As a result, PD-1 and its ligand, PD-L1, are members of the immune checkpoint pathway. Also, inhibitors of PD-1 and PD-L1 may lead to long-term remission in a variety of end-stage malignancies [18]. Immune checkpoint inhibitors have already been the breakthroughs of cancer immunotherapy [19]. Hence, it is thought that inhibiting PD-L1 expression in the tumor microenvironment might have therapeutic implications.

In this study, we sought to gain insight into the level of m5C methylation modifications of lncRNAs in UCEC. To this end, we performed global mapping of m5C modifications in human UCEC tissues and control tissues using RNA MeRIP-seq to understand their distribution and expression. We found a significantly higher level of m5C modification in UCEC compared to normal controls. This difference could be expressed as intratissue consistency and intertissue variation. Our findings may provide new insights into the epigenetic regulation of m5C of lncRNAs in UCEC and offer directions to the development of new therapeutic approaches for UCEC.

## 2. Methods

### 2.1. Collection of Data

The RNA sequencing (RNAseq) fragments per kilobase million (FPKM) data of UCEC samples were downloaded from The Cancer Genome Atlas (TCGA) database, which were further converted to log2 values. We obtained a total of 552 UCEC tissues and 23 control tissues. Also, clinical characteristics of UCEC patients, which included age, tumor grade, and clinical survival status, were also obtained from the same online database.

### 2.2. Identification of m5C-Associated LncRNAs

Based on previous articles, we summarized a total of 17 regulatory factors associated with m5C RNA methylation (Table 1) [20, 21]. To obtain the expression levels of lncRNAs and 17 m5C methylation regulators in TCGA-UCEC mRNA expression profiles, we used the “igraph” package in the R program to construct a coexpression network. The lncRNAs in this coexpression network were significantly correlated with the m5C methylation regulators and were therefore defined as m5C-associated lncRNAs.

### 2.3. Identification of m5C-Associated LncRNAs with Prognostic Value

We then explored the prognostic value of these obtained m5C-associated lncRNAs using the “survival” package in the R program. Based on the results from the univariate Cox analysis, we generated a forest plot using the R program. In addition, by using the “pheatmap” package, we constructed a heat map which adequately presented the expression levels of those 32 lncRNAs with significant prognostic value.

### 2.4. Bioinformatics Analysis

We used the “ConsensusClusterPlus” package to classify 552 UCEC patients into different subtypes. Then, we evaluated and visualized the gene expression patterns using the “pheatmap” package. Using the ESTIMATE algorithm, we calculated the immuneScore, stromalScore and ESTIMATESScore for each UCEC patient. The infiltration abundance of 22 types of immune cells in each UCEC subtype was visualized using the “vioplot” package. A risk model with prognostic value based on m5C-associated lncRNAs was developed by LASSO regression analysis. Risk scores for all UCEC patients were calculated in the TCGA training and testing sets. Subsequently, the median score of UCEC patients in the TCGA training set was set as the cut-off point, based on which patients in each set were divided into high-risk and low-risk groups. In addition, we analyzed the correlation between risk scores and immune cell abundance.

### 2.5. Statistical Analysis

All statistical tests were performed with R version 4.0.1. Student's *t*-test was also used to assess the differences between subgroups. And, the Mann-Whitney *U* test was used to investigate mRNA expression levels of lncRNAs related to m5C. While a chi-square test was used to compare categorical variables in the TCGA training and testing sets. Using Pearson correlation tests, we explored correlations between subtypes, clinicopathological characteristics, risk scores, and immune infiltration levels. *P* < 0.05 indicates statistical significance.

## 3. Results

### 3.1. Identification of LncRNAs Related to m5C in UCEC Patients

**Figure 1:**
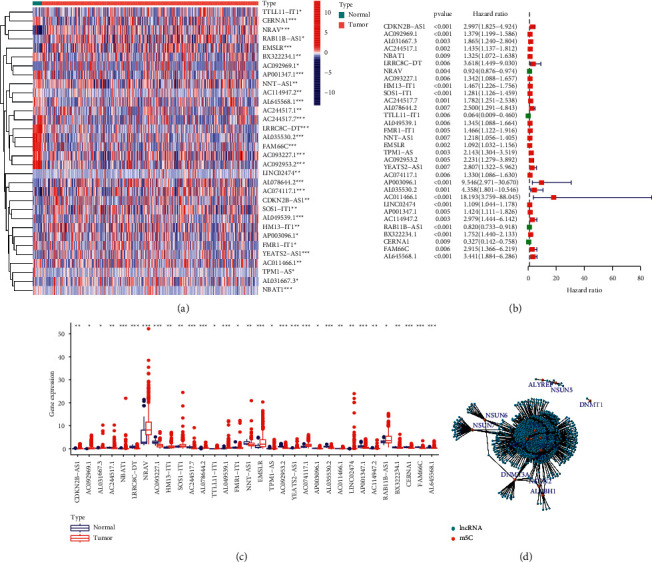
Identification of lncRNAs related to m5C in UCEC patients. The heatmap (a) of 32 m5C-related prognostic lncRNAs and their expression levels (b) in TCGA-UCEC dataset. (c) The univariate Cox regression analysis results on the 32 prognostic lncRNAs. (d) The coexpression network of m5C regulators and 32 identified m5C-related lncRNAs.

To assess the biological functions of m5C-associated lncRNAs in UCEC, we downloaded the expression profile information from the TCGA-UCEC dataset containing a total of 575 samples and extracted expression matrices for 17 m5C regulators. We defined lncRNAs, whose expression values correlated with one or more of the 17 m5C regulators, as m5C-associated lncRNAs. Using Pearson correlation analysis, we obtained a total of 844 lncRNAs significantly associated with m5C regulators. Combined with UCEC clinical characteristics, we screened m5C-associated lncRNAs from these 844 selected lncRNAs by univariate Cox regression analysis (*P* < 0.01). Finally, we found that 32 lncRNAs were significantly correlated with overall survival (OS) of patients with UCEC. The differences in the 32 prognostic m5C-associated lncRNAs levels between UCEC and normal tissues were significant (Figures 1(a) and 1(b)). The univariate Cox analysis results and the coexpression network of the 32 selected lncRNAs and m5C regulators were shown in Figures 1(c) and 1(d). All these results suggested that m5C-related lncRNAs played important roles in patients with UCEC.

### 3.2. Correlation of Consensus Clustering of m5C-Associated LncRNAs with Clinical Characteristics and Survival of UCEC Patients

**Figure 2:**
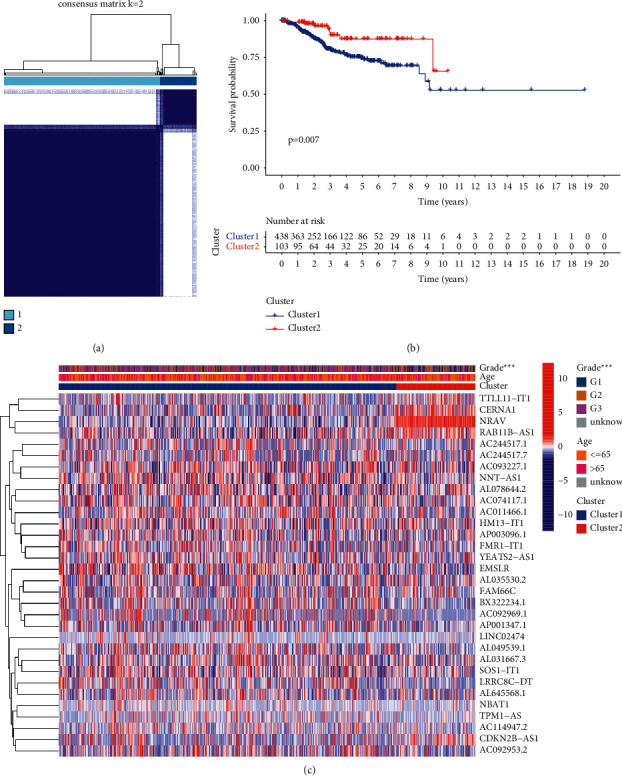
Consensus clustering of m5C-related lncRNAs with prognostic value. (a) Consensus clustering matrix for *k* = 2 in TCGA-UCEC dataset. (b) Kaplan-Meier curves of OS for UCEC patients in two clusters (cluster 1/2). (c) The heatmap of the two clusters in UCEC along with clinical characteristics.

Based on the expression levels of 32 m5C-related lncRNAs, we carried out consensus clustering to divide the 552 samples in TCGA-UCEC into different subgroups and determined the optimal clustering stability with *k* = 2. As shown in Figure 2(a), the 552 UCEC samples were divided into cluster 1 and cluster 2. It was obvious that the expression levels of m5C-related lncRNAs were lower in cluster 1 than in cluster 2. Also, the OS of UCEC patients in cluster 1 was longer than that in cluster 2 (Figure 2(b), *P* = 0.007). In addition, the heatmap of the two clusters in UCEC along with clinical characteristics was shown in Figure 2(c).

### 3.3. Relationship between the Expression Levels of PD-L1 and m5C-Related LncRNAs

**Figure 3:**
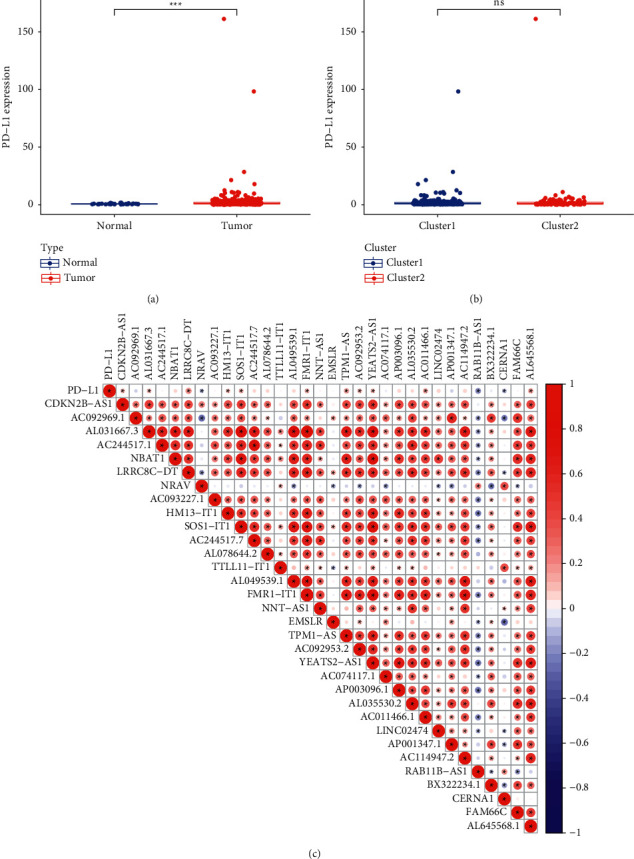
Association of PD-L1 with m5C-related lncRNAs. (a) The expression levels of PD-L1 in normal and tumor tissues of TCGA-UCEC set. (b) The expression levels of PD-L1 in two clusters. (c) The correlation of PD-L1 and the 32 m5C-related lncRNAs.

To clarify the relationship between PD-L1 and lncRNAs related to m5C, we compared the differences in PD-L1 expression levels between UCEC and controls (Figure 3(a)) and between the two clusters (Figure 3(b)). The results showed that PD-L1 levels were significantly elevated in the UCEC tissues (*P* < 0.001). As shown in Figure 3(c), the expression level of PD-L1 was closely associated with the expression level of 14 of the 32 identified lncRNAs. In addition, the selected prognostic m5C-related lncRNAs were positively correlated with each other.

### 3.4. Relationship of Immune Cell Infiltration and Consensus Clustering in UCEC

**Figure 4:**
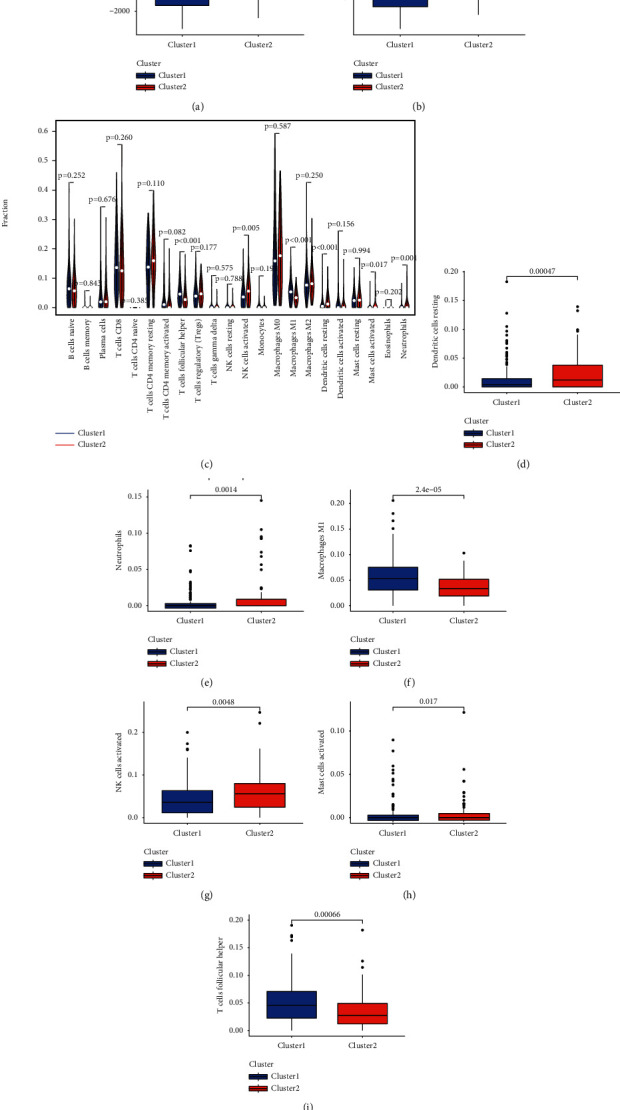
The relationship of immune cell infiltration and consensus clustering in UCEC. (a, b) ImmuneScore and ESTIMATEScore in clusters in UCEC. (c) The infiltrating levels of 22 immune cell types in two clusters in TCGA-UCEC. (d–i) The infiltrating levels of the resting dendritic cells (d), neutrophils (e), macrophages M1 (f), activated NK cells (g), activated mast cells (h), and T cells follicular helper (i) in two clusters.

To explore the roles of m5C-associated lncRNAs in the immune microenvironment of UCEC tumors, we analyzed the differences in immune scores and immune cell infiltration levels between the two clusters. Both ESTIMATEScore (Figure 4(a)) and immuneScore (Figure 4(b)) in cluster 1 were lower than those in cluster 2.  Figure 4(c) showed the infiltration abundance of 22 immune cell types in both clusters. Compared with cluster 2, as shown in Figures 4(d)–4(i), cluster 1 had higher infiltration abundance of macrophages M1, activated mast cells, and T-cell follicular helper cells and lower abundance of resting dendritic cells, neutrophils, and activated NK cells.

### 3.5. Construction and Validation of Prognostic Risk Model Based on m5C-Related LncRNAs

**Figure 5:**
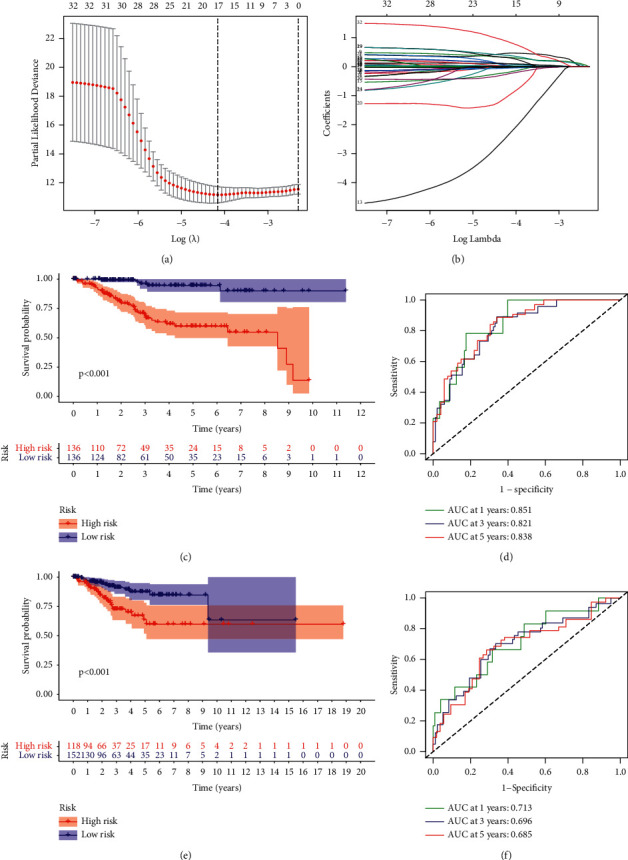
Construction of the risk model according to the identified m5C-related lncRNAs in UCEC. (a, b) LASSO regression and corresponding cross-validation of 32 m5C-related lncRNAs with prognostic value in UCEC. (c–f) OS analysis for UCEC patients and the ROC curve for measuring the corresponding predictive values in TCGA training set (c, d) and TCGA testing set (e, f).

A total of 552 TCGA-UCEC patients were randomly grouped into two sets, a training set and a testing set. We carried out LASSO Cox analysis of the identified 32 m5C-related lncRNAs with prognostic value using information extracted from the training set. The results and partial likelihood deviations of the prognostic risk model were shown in Figures 5(a) and 5(b), while corresponding coefficients of the lncRNAs were shown in Table 2. The risk score for each UCEC patient was calculated using the constructed signature: score = ∑_*i*=1_^*n*^coef(*i*)*∗*  exp(*i*). The UCEC samples in both sets were then divided into high-risk group or low-risk group based on the median score of the training set. And the OS curves showed that patients with UCEC in the high-risk group had worse prognosis in both the training set (Figure 5(c)) and the testing set (Figure 5(e)). As shown in Figure 5(d), the AUC was, respectively, 0.851, 0.821, and 0.838 at 1, 3, and 5 years, indicating good performance of this risk model in predicting the clinical prognosis of UCEC patients in training set. Data from the testing set further confirmed the predictive value of the m5C-related risk signature (Figure 5(f)).

**Figure 6:**
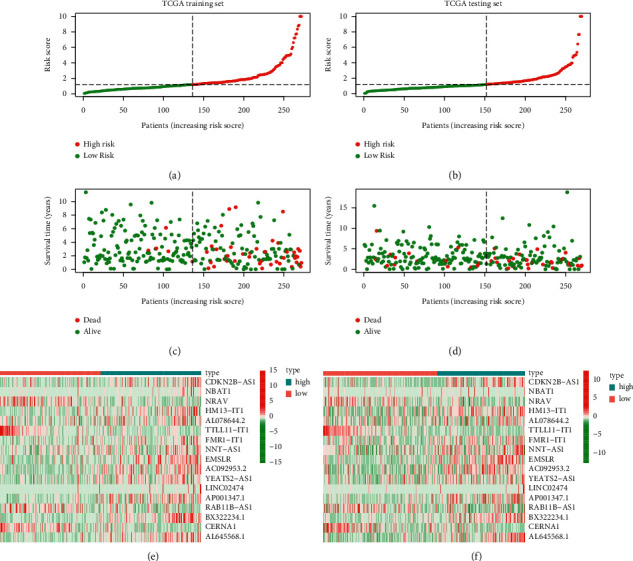
Validation of the constructed risk model based on the m5C-related lncRNAs in UCEC. (a and b) Distribution of risk scores of UCEC samples in TCGA training set (a) and TCGA testing set (b). (c, d) Survival status of samples in two sets. (e, f) Heatmaps of the m5C-related lncRNAs expression levels in two TCGA sets.

Figures 6(a)–6(d) showed the distribution of risk scores and survival status of patients with UCEC in the training and testing sets. The mRNA expression levels of lncRNAs including NBAT1, NRAV, TTLL11-IT1, FMR1-IT1, YEATS2-AS1, RAB11B-AS1, and CERNA1 were lower in the UCEC samples with higher risk scores, while the other 10 m5C-related lncRNAs were lower in the low-risk group (Figures 6(e) and 6(f)).

### 3.6. Relationship of Prognostic Risk Score with Clustering Subtypes and Clinical Factors

**Figure 7:**
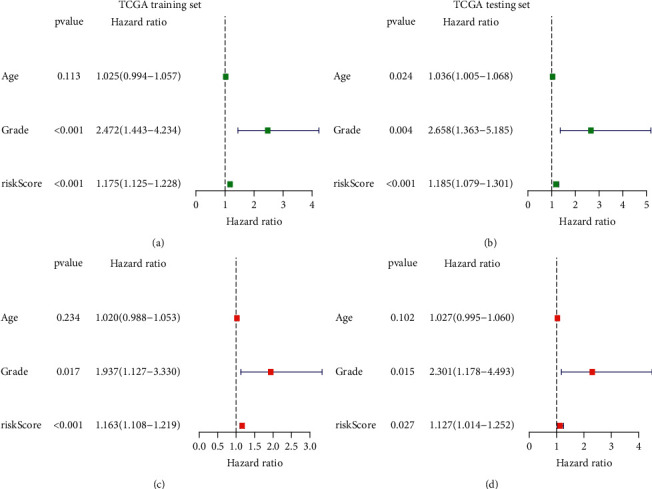
Independent validation of m5C-related lncRNAs-based risk model along with clinical features. Univariate and multivariate Cox regression analyses considering riskScore and clinical features in the training set (a, b) and the testing set (c, d).

To further validate the prognostic value of the risk model in UCEC, we carried out univariate and multivariate Cox regression analyses on risk scores and clinical information of UCEC patients. The results showed that both tumor grade and risk model were significantly associated with the clinical prognosis of patients with UCEC in the training set (Figure 7(a)). The results of multivariate Cox regression analysis further indicated that the risk model was an independent factor with predictive value affecting the clinical outcome of UCEC patients (Figure 7(b)). Similarly, the results of univariate and multivariate Cox regression analyses in the testing set were consistent with those in the training set (Figures 7(c) and 7(d)).

**Figure 8:**
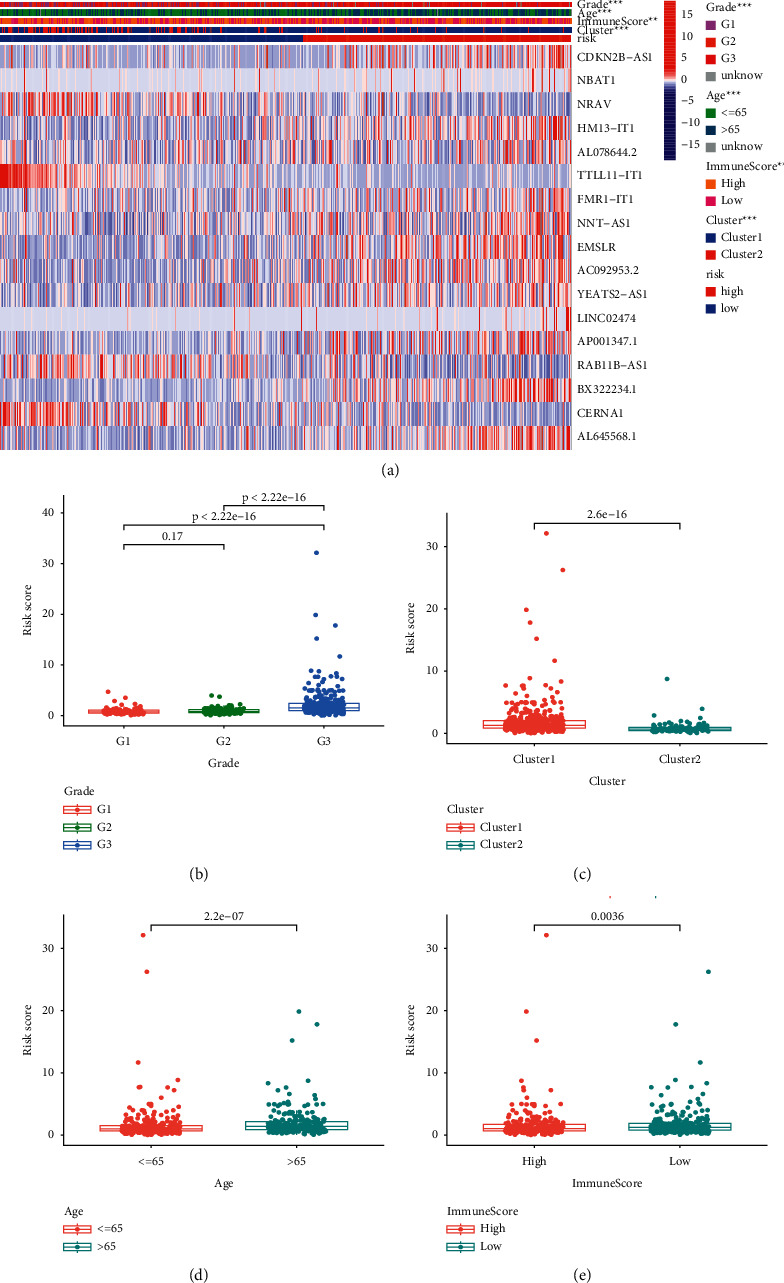
The relationship of risk score with clinical features and immuneScores in patients with UCEC. (a) Heatmap of m5C-related lncRNAs expressions and clinical features between two risk groups. (b–e) The risk score in different tumor grade (b), cluster (c), age (d), and immuneScore (e) of patients with UCEC.

The heatmap in Figure 8(a) showed the mRNA expression levels of the 17 lncRNAs, which were used to establish the risk model, in the two risk groups in the entire TCGA-UCEC dataset. The heatmap also showed the differences between high- and low-risk groups in terms of tumor grade, age, immune score, and cluster subtypes. More specifically, risk scores were lower in the low-grade (grades I and II) UCEC group compared with the high-grade (grade III) group (Figure 8(b)). Similarly, the risk score was higher when the age of the UCEC patients increased (age >65) (Figure 8(d)). The results also showed that the risk scores differed between the two clusters (Figure 8(c)). The risk scores were statistically different between the two risk groups (Figure 8(e)).

### 3.7. Linkage between m5C-Associated LncRNAs and Immune Cells

**Figure 9:**
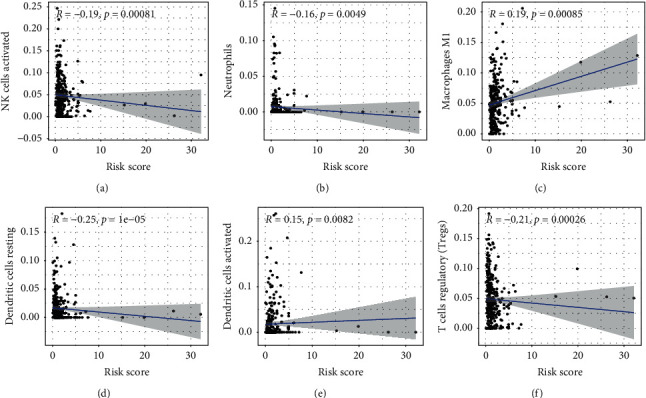
The relationship between the risk score and the immune cell infiltration. (a-f) NK cells activated (a), neutrophils (b), macrophages M1 (c), dendritic cells resting (d), dendritic cells activate (e), and T cells regulatory (f).

To explore the impact of risk models constructed based on m5C-related lncRNAs on the immune microenvironment of UCEC, we explored the potential relationship between the risk scores and the immune cell infiltration in UCEC patients. The results showed a significant negative correlation between risk score and the infiltration levels of activated NK cells (Figure 9(a)), neutrophils (Figure 9(b)), resting dendritic cells (Figure 9(d)), and regulatory T cells (Figure 9(f)). In contrast, the risk scores were positively related to the infiltration levels of M1 macrophages (Figure 9(c)) and activated dendritic cells (Figure 9(e)). All these results confirmed that this risk model was closely related to the immune microenvironment of UCEC.

## 4. Discussion

Several recent studies have shown that m5C regulators play important roles in tumor progression by regulating the expression and function of lncRNAs [22, 23]. For example, the m5C reader, NSUN2, promotes hepatocellular carcinogenesis and progression by regulating the m5C modification of lncRNA H19 [24]. However, there are no studies that have adequately explored the role of m5C-related lncRNAs in the clinical prognosis and immune microenvironment of UCEC. In this study, we comprehensively explored the expression levels and the prognostic values of m5C-associated lncRNAs in UCEC and their effects on the immune microenvironment. First, we identified m5C-related lncRNAs by constructing a coexpression network along with the survival status of UCEC patients. As a result, 32 m5C-related lncRNAs were found significantly associated with survival outcomes in UCEC patients compared with normal tissues. The expression levels of all these lncRNAs were up- or downregulated in UCEC tissues. Consensus clustering analysis of these 32 m5C-associated lncRNAs with prognostic value identified two subtypes of UCEC. It was obvious that the stratification of two cluster subtypes showed a significant correlation with clinical outcome, age, and tumor grade in UCEC patients. PD-L1 is often upregulated in various cancers. Compared with normal tissue, PD-L1 expression was significantly increased in UCEC, but no significant difference was detected between two clusters. We calculated the immuneScore and ESTIMATEScore of the UCEC samples and found that both of them in cluster 2 were significantly higher than those in cluster 1. These results were consistent with previous findings that UCEC patients with high immuneScores and ESTIMATEScores have lower overall survival rates [25].

LASSO Cox analysis was carried out on 32 m5C-associated lncRNAs with prognostic value, which finally identified 17 m5C-associated lncRNAs. CDKN2B-AS1 is a potential lncRNA that has been shown to be aberrantly expressed in various malignancies and involved in the processes of tumor cells proliferation, migration, invasion, and inhibition of tumor cells apoptosis [26–28]. NBAT1 could suppress metastasis [29] and control tumor progression by regulating cell proliferation and neuronal differentiation [30]. NRAV modulates antiviral responses through suppression of interferon-stimulated gene transcription [31]. The downexpression of FMR1-IT1 has been found related to synaptogenesis, intracellular trafficking, and cellular stability [32]. The other identified m5C-related lncRNAs are also reported related with oncogenesis and progression of tumors [33–39]. However, so far, little research is carried out on AL078644.2, EMSLR, AC092953.2, and AP001347.1. Future research should focus on such lncRNAs and their roles within UCEC. Then, a risk model was constructed based on the coefficients and the expression levels of the 17 lncRNAs. Based on the calculation results of the risk model, the UCEC samples were divided into high-risk group or low-risk group. We then found that UCEC samples in the group with high risks had poorer prognosis, indicating that this risk model based on m5C-associated lncRNAs had a good performance in predicting the clinical prognosis of patients with UCEC. Moreover, results of univariate and multivariate Cox regression analyses showed that this risk model could serve as an independent predictive factor in UCEC. We further explored the association between the prognostic risk model and the clinical features of UCEC patients. In addition, UCEC patients with grade III had a higher risk score compared with others with grade I or II. Interestingly, the risk scores were significantly higher in the group with high immune scores than in the other group, which was consistent with previous findings that UCEC patients with high immune scores had lower OS [40].

It is well known that the tumor immune microenvironment often plays an important role during the development and progression of cancers [41, 42]. Heterogeneity of the immune microenvironment can influence a variety of factors, including patient response to therapy and clinical prognosis [43, 44]. Previous findings suggest that immune cell infiltration can regulate the progression and metastasis in patients with cancers [45, 46]. Another important finding of this study was that the constructed risk score was closely related to the level of immune cell infiltration, which mainly included activated NK cells, neutrophils, resting dendritic cells, regulatory T cells, M1 macrophages, and activated dendritic cells. Notably, there was a significantly negative association between the risk score and the levels of regulatory T-cell infiltration.

This study also has several limitations. First, our results were obtained by analyzing data from TCGA. Other databases such as GEO are needed to further validate the validity of this risk model. Meanwhile, we need to conduct clinical and basic experiments to further validate the predictive value of the constructed risk model. Second, the potential mechanisms of the regulation of these m5C-related lncRNAs in UCEC deserve further investigation.

In conclusion, our study systematically explored the prognostic value of m5C-related lncRNAs and their effects on the immune microenvironment and clinical outcomes in UCEC via consensus clustering of them and constructing a risk model with predictive value. The results in this study suggest that m5C-related lncRNA-based risk models may serve as an important mediator of the immune microenvironment in UCEC. Our findings provide a potential theoretical basis for future clinical studies that use the m5C-related lncRNAs as promising therapeutic targets for UCEC.

## Figures and Tables

**Table 1:** Details of m5C RNA methylation regulators.

Gene	Type
DOP2	Writers
NSUN2	Writers
NSUN3	Writers
NSUN4	Writers
NSUN5	Writers
NSUN6	Writers
NSUN7	Writers
DNMT1	Writers
TRDMT1	Writers
DNMT3A	Writers
DNMT3B	Writers
ALYREF	Readers
YBX1	Readers
TET1	Erasers
TET2	Erasers
TET3	Erasers
ALKBH1	Erasers

**Table 2:** Results of LASSO Cox analysis on the 32 identified m5C-related lncRNAs.

Gene	Coef
CDKN2B-AS1	0.469698
NBAT1	−0.01305
NRAV	−0.02457
HM13-IT1	0.321693
AL078644.2	0.03965
TTLL11-IT1	−2.32991
FMR1-IT1	−0.18714
NNT-AS1	0.101763
EMSLR	0.049661
AC092953.2	0.308109
YEATS2-AS1	−0.9855
LINC02474	0.10311
AP001347.1	0.00306
RAB11B-AS1	−0.04677
BX322234.1	0.38926
CERNA1	−0.24199
AL645568.1	0.85146

## Data Availability

The datasets generated and analyzed during the current study are available on TCGA database (http://cancergenome.nih.gov/abouttcga).
